# Brain Magnetic Susceptibility is Associated With Slower Gait in Community-Dwelling Older Adults

**DOI:** 10.1093/geroni/igab046.3177

**Published:** 2021-12-17

**Authors:** Victoria Poole, Robert Dawe, Sue Luergans, David Bennett, Aron Buchman, Konstantinos Arfanakis

**Affiliations:** 1 Rush University, Chicago, Illinois, United States; 2 Rush University, Rush Alzheimer's Disease Center, Illinois, United States

## Abstract

Age-related slowing of gait is exceedingly common and a robust predictor of various adverse health outcomes in older age. Prior neuroimaging studies have documented diverse non-specific structural brain abnormalities which are related to slow gait; however, the extent to which quantitative susceptibility mapping (QSM), which measures regional magnetic susceptibility in the brain, associates with gait speed remains unexplored. In the current study, 415 non-demented community-dwelling older adults (91 males; 81+/- 7 years) underwent an MRI (Siemens 3T TIM Trio) and in-home motor assessment. Gait speed was measured and averaged across 2 timed 8-ft walks. MR-acquired QSM data were pre-processed, registered to ICBM template, and spatially smoothed with a 5mm FWHM Gaussian kernel. When these maps entered group-level GLMs, voxel-wise associations with gait speed were of interest, after adjusting for demographics. We observed very strong negative associations between gait speed and magnetic susceptibility, such that those with slower gait had higher susceptibility in bilateral inferior frontal, superior temporal, and angular gyri (corrected p<.0005). Robust associations were also observed in the middle frontal, precentral, and postcentral gyri of the right hemisphere. These novel findings suggest that reduced myelination or increased iron accumulation in these brain regions may contribute to impaired gait. Future work will need to determine to what extent these cross-sectional QSM metrics are independent predictors of incident adverse health outcomes when controlling for other common brain imaging abnormalities observed in older adults.

